# Viscoelastic Characteristics of Potato Tissue Based on Stress Relaxation Tests

**DOI:** 10.3390/ma19153172

**Published:** 2026-07-24

**Authors:** Zbigniew Stropek

**Affiliations:** Department of Mechanical Engineering and Automation, University of Life Sciences in Lublin, Głęboka 28, 20-612 Lublin, Poland; zbigniew.stropek@up.lublin.pl

**Keywords:** potato, Maxwell model, stress relaxation, viscoelasticity

## Abstract

Stress relaxation tests on cylindrical samples of the Bellarosa potato cultivar were conducted over a wide range of deformation velocities, from 0.0002 m/s to 1.5 m/s. A three-element Maxwell model was applied to describe the response of potato tissue to the applied load. The effect of deformation velocity on the Maxwell model parameters and the values derived from the experimental stress relaxation curves was determined. The viscoelastic behavior of potato tissue was confirmed by the increase in the maximum force response with increasing deformation velocity. The relaxation times *τ*_1_ and *τ*_3_ decreased as the deformation velocity increased. The minimum force recorded at the end of the test and the elastic modulus *E*_3_ were substantially higher under quasi-static than under impact loading conditions. These two parameters provide a good description of the material state after deformation and indicate a greater degree of cellular structure damage during impact loading compared with compression at low deformation velocities. Potato tissue contains approximately 80% water and only 3% air spaces. Therefore, changes in the cellular structure are expected to be associated mainly with water movement within cells and through intercellular spaces, with the intensity of these processes depending on the deformation velocity.

## 1. Introduction

The quality of fruit and vegetables is the result of the complex impact of physical, chemical, and biological properties [1]. However, mechanical parameters most significantly determine the ability of plant materials to sustain the loads related to harvesting, transport, packaging, and storage [2,3]. The modern fresh food market sets high standards for reducing quality and quantity losses, which is why methods that enable quantitative assessment of the product susceptibility to mechanical damage are becoming increasingly important. One of the most valuable measurement techniques is stress relaxation tests, commonly used to test materials with viscoelastic properties [4,5,6].

Stress relaxation results in a gradual decrease in stress in a material at a constant deformation. After deformation to a certain value, the initial stress value reaches a maximum level and then gradually decreases in time. For biological materials, the speed of this decrease depends on factors such as cell structure, water content, degree of ripeness, temperature, and storage conditions [7].

Fruit and vegetable tissues have a complex cellular structure, characterized by the presence of water, cell membranes, pectins, and supporting elements such as cellulose and hemicellulose [8]. This structure determines the behavior of the material under load—fruit and vegetables are not typical solids or liquids but exhibit characteristics of both states.

In order to quantitatively describe the phenomenon of stress relaxation, viscoelastic models are used, which describe the behavior of materials exhibiting both elastic and viscous properties. The most commonly used models are based on mechanical elements such as a spring (elastic element) and a dashpot (viscous element), which allow for a mathematical representation of stress changes in time. One of the simplest and most commonly used models is the Maxwell model, consisting of a spring connected in series with a dashpot.

The Maxwell model assumes that under sudden deformation, the stress initially reaches a maximum value and then decreases exponentially in time as a result of fluid flow in the material structure or changes in the arrangement of cellular elements. In relation to fruit and vegetables, this allows determining how quickly the tissue “disperses” the energy of deformation, which is directly related to their firmness and resistance to mechanical damage [9]. Model parameters such as elastic modulus and dynamic viscosity allow for comparison of different loading directions, degrees of ripeness, as well as assessing the effect of storage conditions on the mechanical properties or quality of the product [10,11].

In some cases, more complex models, such as Kelvin–Voigt [12] or Burgers [13,14], which combine elastic and viscous elements in more extensive systems, are also used to describe the phenomenon more accurately. This makes it possible to describe both the immediate elastic response and the long-term relaxation processes typical of tissues rich in water and pectin substances. However, despite the greater complexity of multi-parameter models, the Maxwell model remains one of the basic tools for analyzing stress relaxation in plant product research due to its simplicity of interpretation and ease of fitting to experimental data.

The aim of this article was to determine the Maxwell model parameters characterizing potato tissue under compressive loading and to investigate the effect of deformation velocity on both the Maxwell model parameters and the characteristics derived from the experimental stress relaxation curves.

## 2. Materials and Methods

### 2.1. Material

The study was conducted on cylindrical samples of “Bellerosa” potatoes. The samples were 21 mm in height and 15 mm in diameter. Three cylindrical samples were obtained from each potato to perform stress relaxation tests at three different deformation velocities. This sampling approach was adopted to reduce variability in the measurement results and to evaluate the mechanical response of tissue from the same potato under different deformation velocities. The same procedure was applied under both quasi-static and impact loading conditions.

The cylindrical samples were prepared using a cutter with an internal diameter of 15 mm. Each sample was then placed in a 21 mm high cutting sleeve and trimmed to the required height.

### 2.2. Stress Relaxation Tests

Cylindrical samples were initially compressed by 3 mm along the vertical axis under uniaxial loading conditions. The deformation was then maintained at a constant level while the force response was recorded for 30 s. The test duration was selected because only minor changes in the force response were observed after the first 30 s.

The initial deformation of 3 mm was determined based on preliminary quasi-static compression tests. It was assumed that this deformation corresponded to a force equal to approximately 50% of the force required to cause sample failure. All tests were carried out at room temperature. Stress relaxation experiments were performed at six deformation velocities. Under quasi-static loading conditions, the tests were carried out using a TA.HD Plus texture analyzer at deformation velocities of 0.0002, 0.002, and 0.02 m/s. Under impact loading conditions, the experiments were conducted using the impact testing stand described in [15] at deformation velocities of 0.5, 1.0, and 1.5 m/s.

Ten repetitions were performed for each deformation velocity, resulting in a total of 60 stress relaxation tests.

### 2.3. Measuring Device

**Figure 1:**
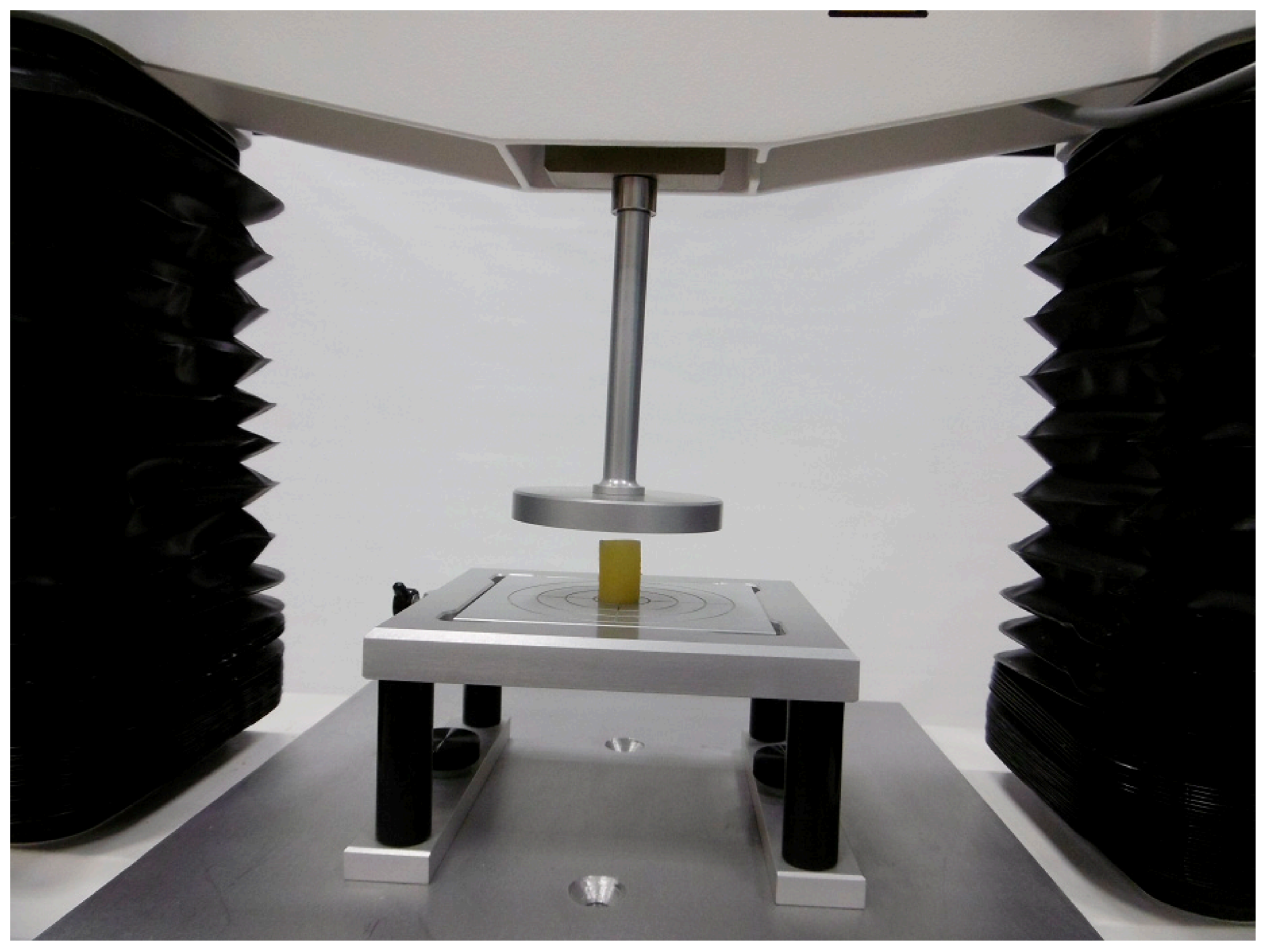
TA.HD plus texture analyzer.

The tests under quasi-static loading conditions were performed on a TA.HD plus two-column texture analyzer (Stable Micro Systems company, Goldaming, UK) equipped with a 300 N load cell (Figure 1). The data acquisition frequency was set to 100 Hz. Force recording was initiated automatically when the measured force exceeded 0.1 N.

**Figure 2:**
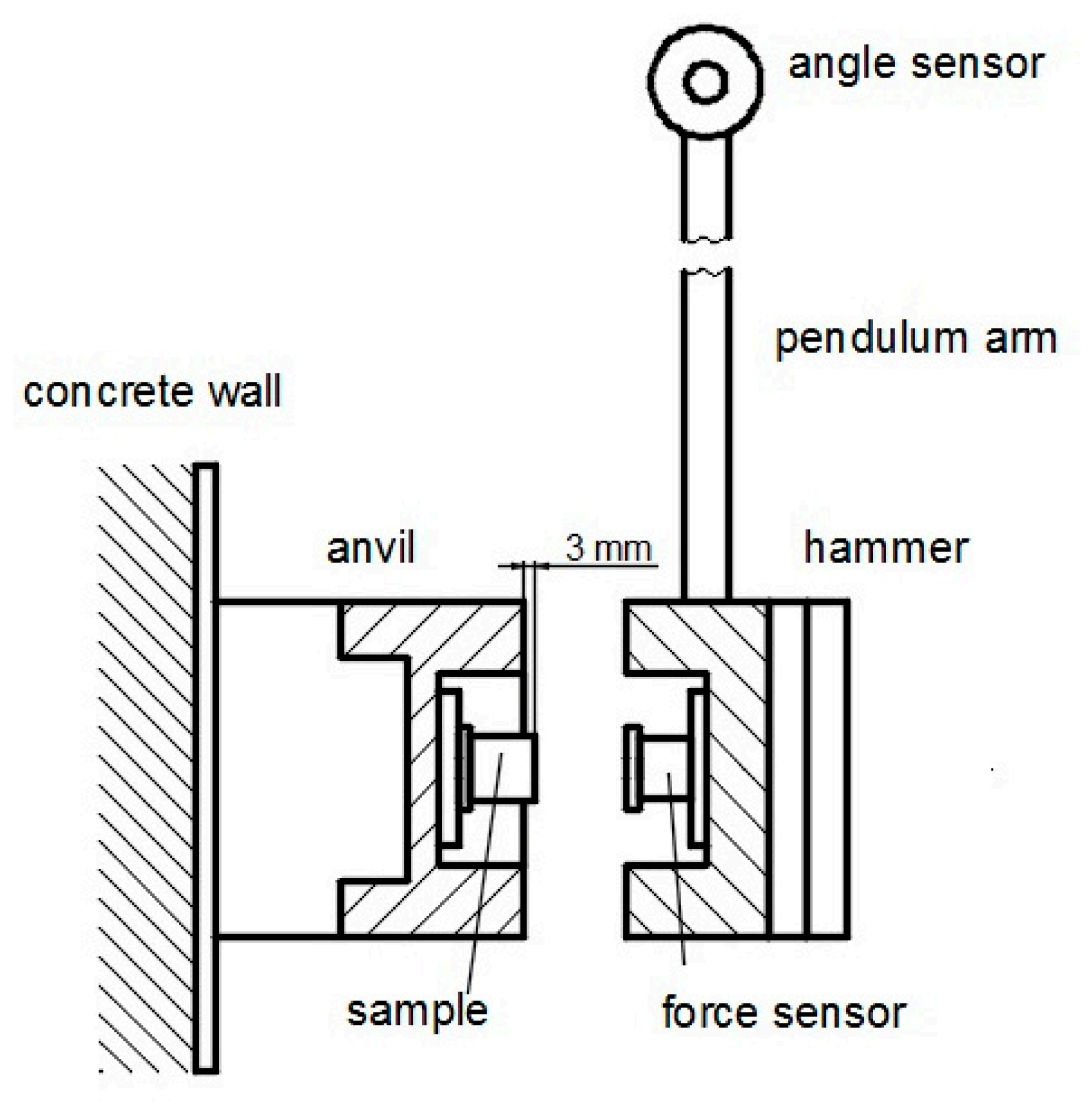
Impact measuring stand.

A pendulum with a rigid arm ended with a cylindrical hammer, in which a piezoelectric force sensor was placed, was used in the tests of cylindrical samples under impact loading conditions (Figure 2). The hammer mass was selected so that its kinetic energy, even at the lowest drop height, was substantially greater than the deformation energy of the sample. Under these conditions, the deformation process could be assumed to occur at an approximately constant velocity. The pendulum arm was 940 mm long, and the hammer displacement during contact with the sample was limited to 3 mm. Consequently, the deformation of the sample was assumed to occur along a straight-line path.

The cylindrical anvil was mounted to a vertical concrete wall. Each cylindrical sample was attached with technical Vaseline to a vertical plate screwed into the anvil. A non-contact angular position sensor (WMU45SK, WOboit, Pniewy, Poland) was mounted on the pendulum axis to measure the angular displacement of the pendulum arm relative to the vertical. These measurements were used to determine both the drop height and the impact velocity.

The impact force was measured using a piezoelectric force sensor (Endevco, Sunnyvale, CA, USA), model 2311-100, with a sensitivity of 23.17 mV/N and a measuring range of ±220 N. The sensor converts the electrical charge generated by the piezoelectric element into a voltage signal proportional to the applied force. The sensor output was recorded using a microprocessor data acquisition system, which transmitted the data to a computer. The sampling frequency of the data acquisition system was 1.05 kHz.

### 2.4. Experimental Curves Modeling

To approximate the experimental stress relaxation curves, 33 measurement points were selected, with the first 10 for every 0.01 s, the next 10 for every 0.1 s, the next 9 for every 1 s intervals, and the last 4 for every 5 s. This non-uniform sampling method was adopted to reflect the time-dependent changes in the force response during stress relaxation, where rapid force decay occurs immediately after loading, followed by a much slower decrease. To automate the selection of these measurement points, a dedicated macro was developed in Microsoft Excel.

The selected data points were fitted using exponential functions of the form:

(1) $$F(t)=\sum_{i=1}^{3}A_{i}\cdot e^{-\alpha_{i}\cdot \left(t-t_{m}\right)}$$

where *A_i_* and *α_i_* are parameters.

The quasi-Newton nonlinear minimization method was used to determine the values of *A_i_* and *α_i_*. The quasi-Newton method consists of estimating the function at various points at each step in order to estimate the first- and second-order derivatives. This was then used to find the minimum of the loss function.

A 3-element Maxwell model was used to describe the stress relaxation curves of potato tissue. The formula given by Chen [16] was used to calculate the force for a cylindrical sample compressed along the axis:

(2) $$F(t)=\left(\frac{S\cdot v}{l}\int_{0}^{t_{m}}\sum_{i=1}^{3}E_{i}\cdot e^{-\frac{E_{i}}{\eta_{i}}\cdot \left(t_{m}-t\right)}\cdot dt\right)\cdot e^{-\frac{E_{i}}{\eta_{i}}\cdot \left(t-t_{m}\right)}$$

where *S* is the cross-sectional area [m^2^], *v* is the deformation velocity [m/s], *l* is the sample length [m], tm is the time when deformation increases [s], t is the time counted from the beginning of the sample deformation [s], *E_i_* is the elastic modulus [Pa], and *η_I_* is the dynamic viscosity [Pa·s]

Formula (2) describes the second phase of the test, in which constant deformation is maintained (t > tm). The formula includes the deformation velocity v, which indicates that the phenomenon of stress relaxation was already taken into account during the period when the sample was deformed.

A comparison of Equations (1) and (2) was used to obtain the values of the model coefficients *E_i_* and *η_i_* [17,18] as described by Equations (3) and (4).

(3) $$E_{i}=\frac{A_{i}\cdot l\cdot \alpha_{i}}{S\cdot v\cdot (1-e^{-\alpha_{i}\cdot t_{m}})}$$

(4) $$\eta_{i}=\frac{A_{i}\cdot l}{S\cdot v\cdot \left(1-e^{-\alpha_{i}\cdot t_{m}}\right)}$$

This made it possible to determine the relaxation time, which is the quotient of *η* and *E*.

(5) $$\tau =\frac{\eta }{E}$$

### 2.5. Moisture Content Measurements

Potato tissue moisture content was also measured in order to refer the results to the texture of the potatoes. Moisture content was determined by drying thin slices (5 mm thick) in a KBC-65W oven (Wamed, Warsaw, Poland) at 80 °C until a constant weight was achieved after 18 h. Ten potatoes were used for the measurements. The weight of the sample was measured before and after drying with an accuracy of 0.1 g. The mean value and standard deviation of the moisture content of the tested potatoes were 81.4 ± 1.8%.

### 2.6. Statistical Analysis

The results were statistically processed using Statistica 13 software (Statsoft, Tulsa, OK, USA). The statistical significance of differences between the mean values of the tested parameters was determined based on one-way analysis of variance (ANOVA) using the LSD test at a significance level of 0.05.

## 3. Results

**Figure 3:**
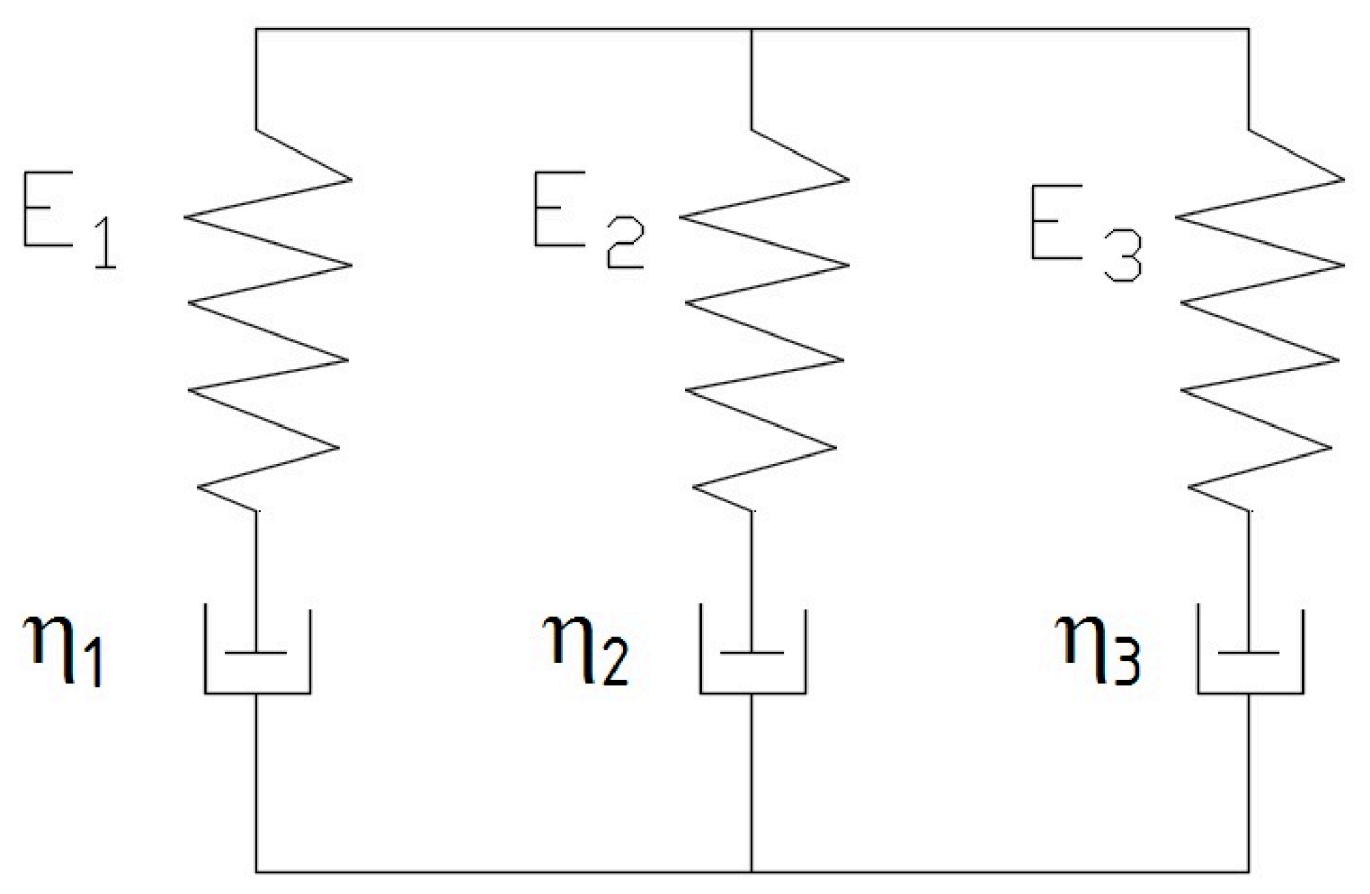
Structure and components of three-element Maxwell model.

The three-element Maxwell model shown in Figure 3 allowed the experimental curve to be divided into three exponential components.

**Figure 4:**
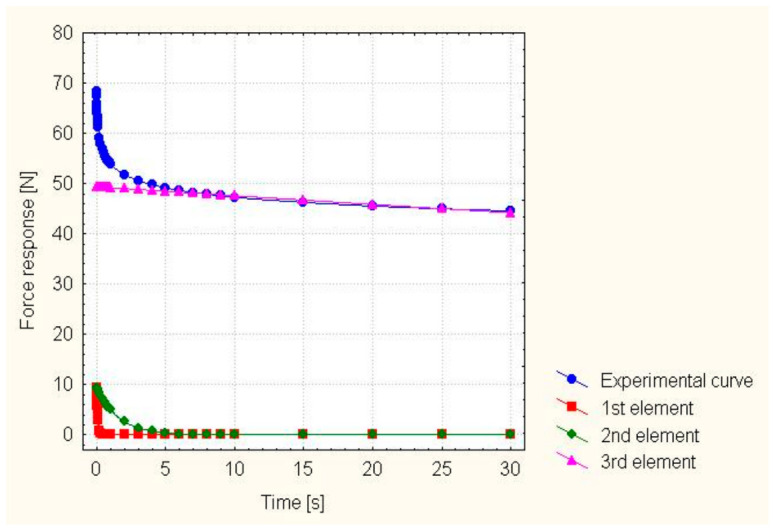
Division of the experimental curve resulting from the assumed Maxwell model.

Figure 4 presents the experimental stress relaxation curve obtained during the test, together with the individual components derived from the assumed Maxwell model.

Figure 4 shows that the first element of the Maxwell model describes the material response during the first 0.1 s of the relaxation process, the second element represents the state of the material over the subsequent 5 s, and the third element characterizes the material response at the end of the relaxation test.

**Figure 5:**
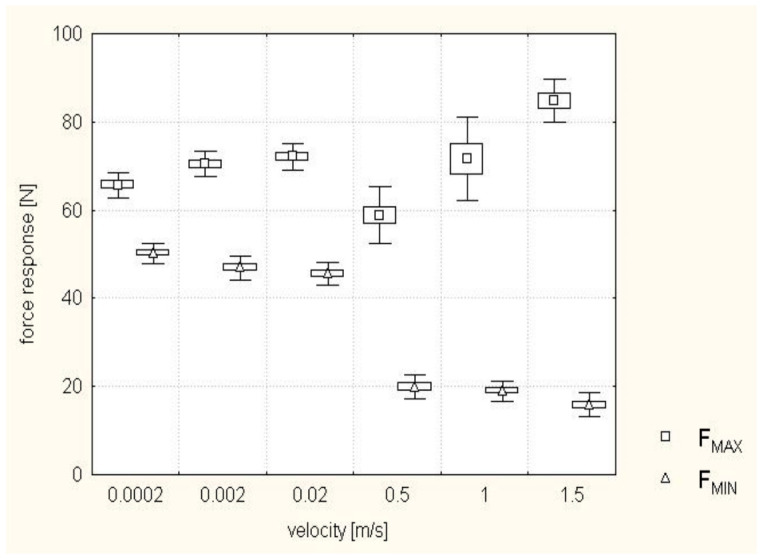
The relationship between maximum and minimum force response and deformation velocity.

The relaxation tests made it possible to determine parameters that can be obtained directly from the experimental curves, namely the maximum force at the beginning of the test and the minimum force at the end of the relaxation test (Figure 5). The maximum force increased with increasing deformation velocity, confirming the viscoelastic nature of potato tissue. In contrast, the minimum force at the end of the relaxation test decreased as the deformation velocity increased. A significant difference was observed between the minimum force values obtained under quasi-static and impact loading conditions. Specifically, the minimum force was substantially higher under quasi-static than under impact loading conditions, indicating more extensive damage to the cellular structure of the potato tissue during impact loading.

**Figure 6:**
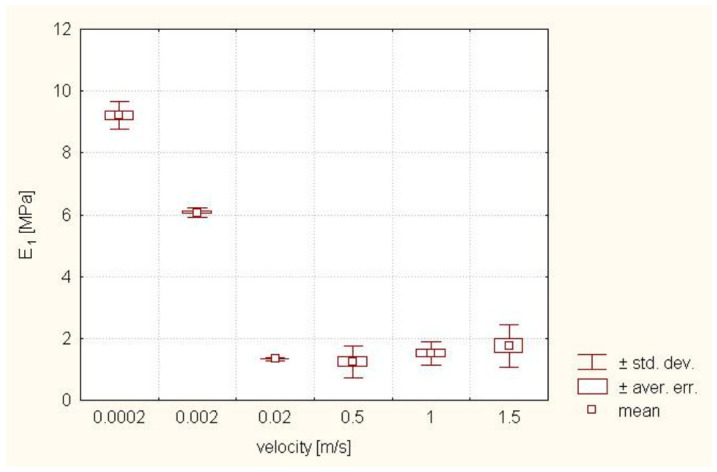
The relationship between elastic modulus *E*_1_ and deformation velocity.

**Figure 7:**
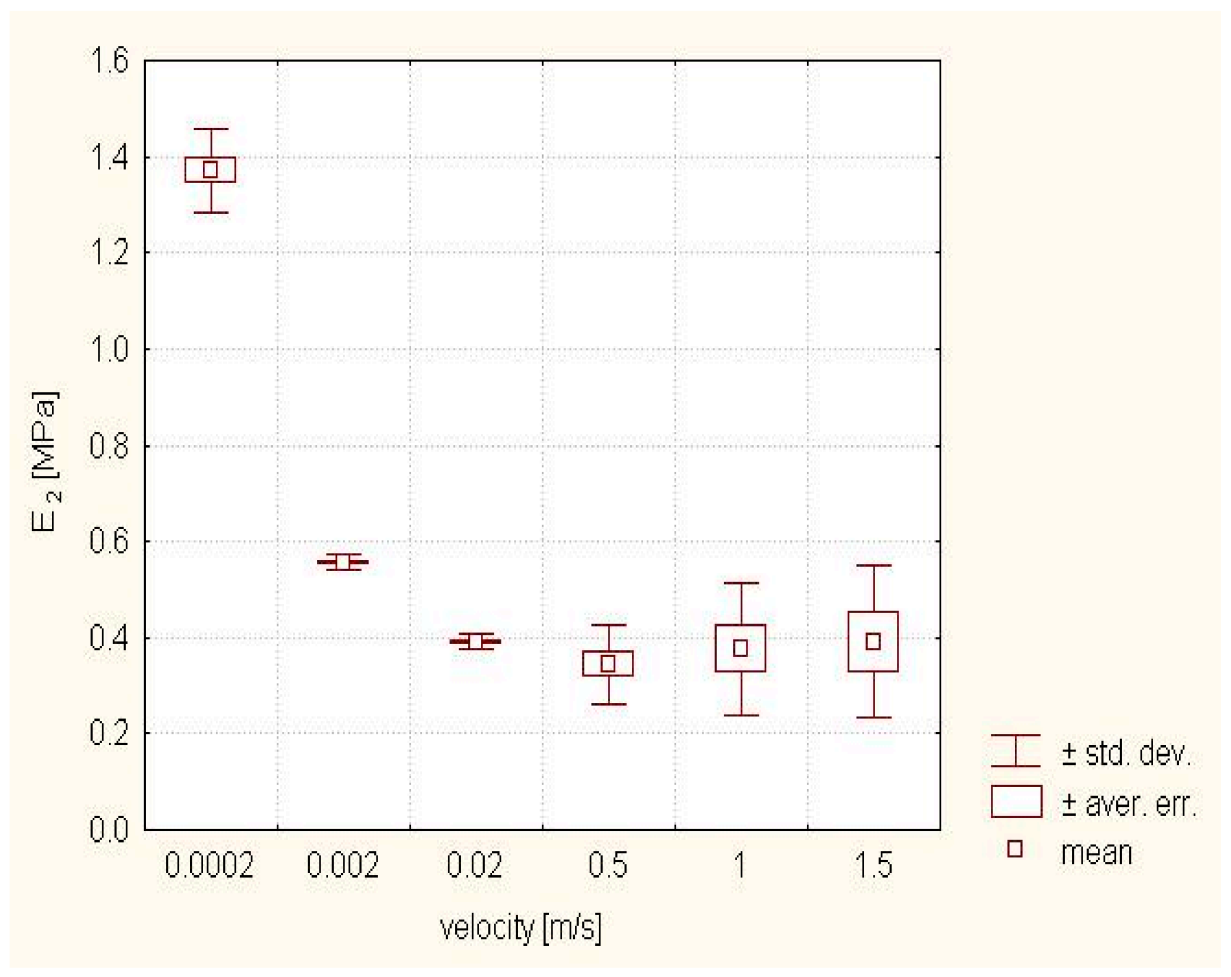
The relationship between elastic modulus *E*_2_ and deformation velocity.

**Figure 8:**
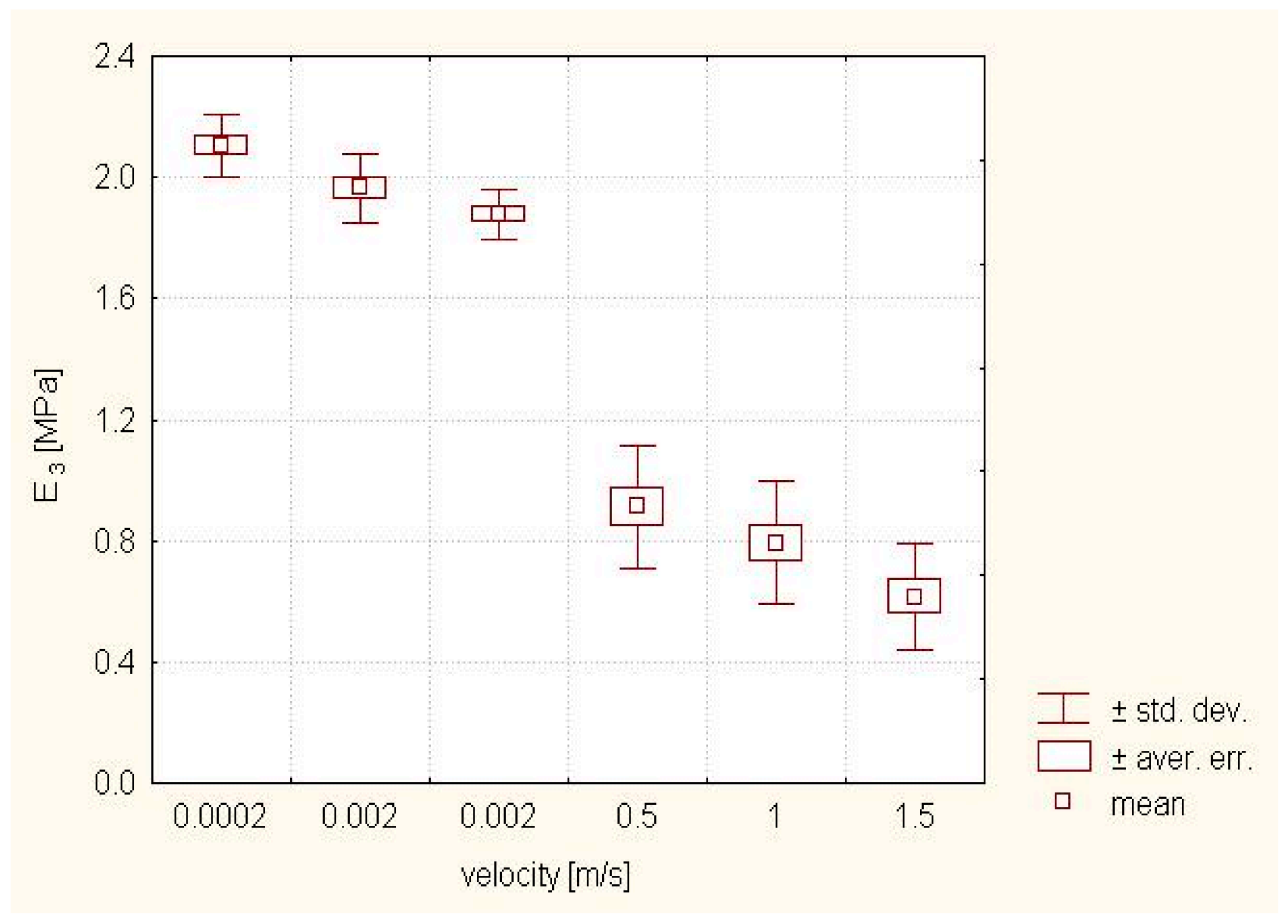
The relationship between elastic modulus *E*_3_ and deformation velocity.

For all three elements of the Maxwell model, the elastic moduli decreased with increasing deformation velocity (Figure 6, Figure 7 and Figure 8). However, the observed trends differed among the individual moduli. The elastic modulus *E*_1_ decreased rapidly and reached a value of approximately 2 MPa at velocities above 0.002 m/s. In the case of *E*_2_, a pronounced decrease was observed between the lowest deformation velocity (0.0002 m/s) and the remaining velocities. For *E*_3_, a clear distinction was found between quasi-static and impact loading conditions, with substantially higher values obtained under quasi-static loading. Since *E*_3_ characterizes the mechanical response of the material at the end of the stress relaxation test, these results may indicate a lower degree of cellular tissue damage under quasi-static loading than impact loading conditions. This interpretation is based on the rheological response of the material and evidence reported in the literature and has not been directly verified by microstructure observations in the present study.

Within both loading regimes, the value of *E*_3_ decreased with increasing deformation velocity.

**Figure 9:**
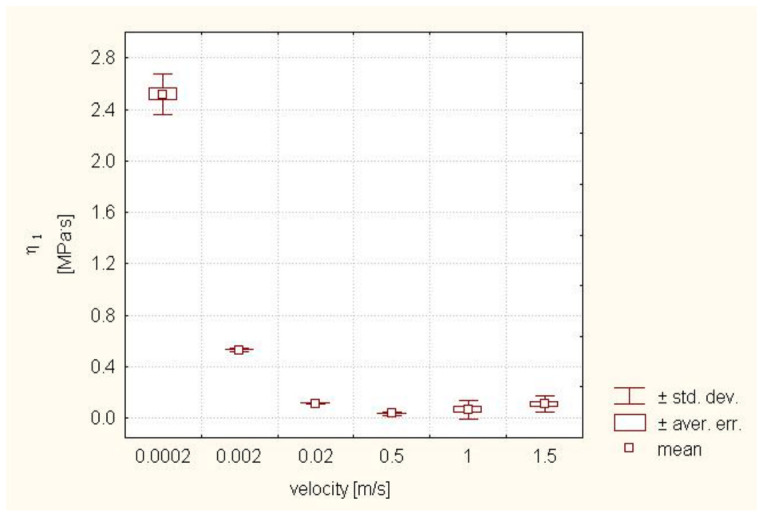
The relationship between dynamic viscosity *η*_1_ and deformation velocity.

**Figure 10:**
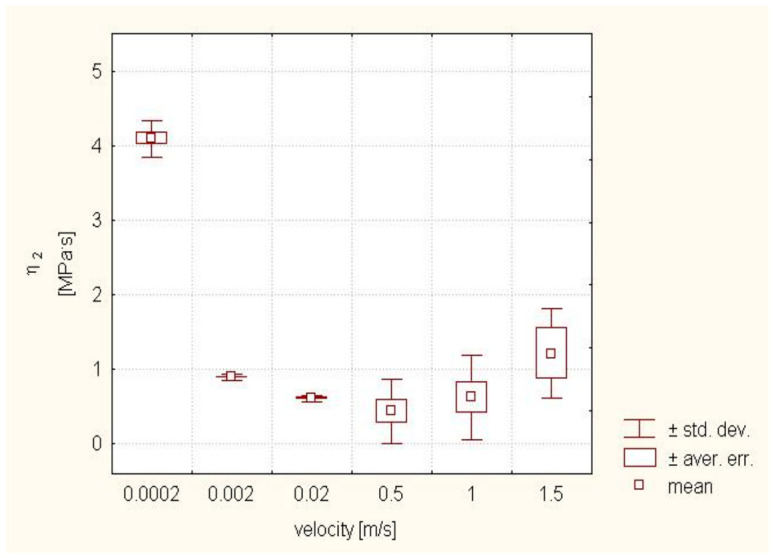
The relationship between dynamic viscosity *η*_2_ and deformation velocity.

**Figure 11:**
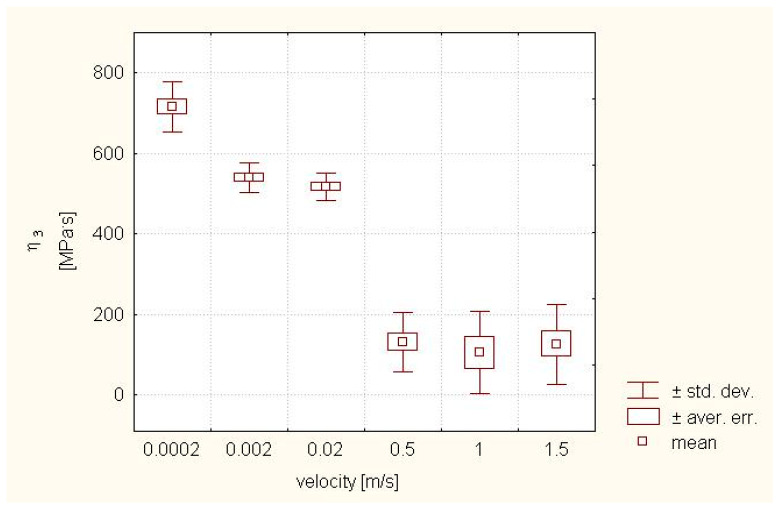
The relationship between dynamic viscosity *η*_3_ and deformation velocity.

The dynamic viscosity of all elements of the Maxwell model decreased with increasing deformation velocity. For the dynamic viscosities *η*_1_ and *η*_2_, a distinct difference was observed between the values obtained at the lowest velocity (0.0002 m/s) and those corresponding to the higher deformation velocities (Figure 9 and Figure 10). In the case of *η*_3_, substantially higher values were observed under quasi-static loading conditions compared with impact loading conditions (Figure 11).

**Figure 12:**
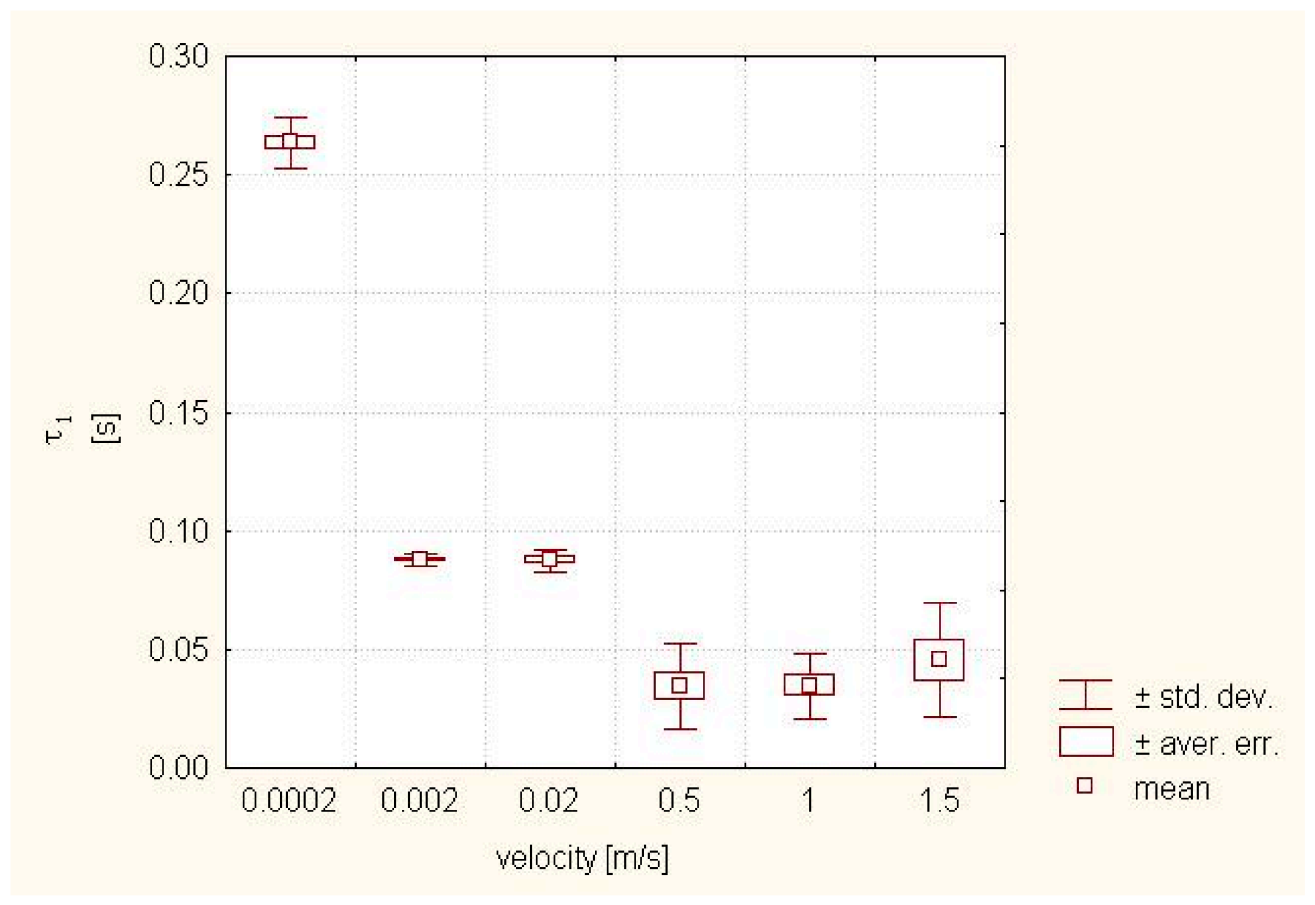
The relationship between relaxation time *τ*_1_ and deformation velocity.

The relaxation times decreased with increasing deformation velocity. Under quasi-static loading conditions, a pronounced decrease in *τ*_1_ values was observed between deformation velocities of 0.0002 m/s and 0.002 m/s. Under impact loading conditions, the *τ*_1_ values did not exceed 0.05 s, and no statistically significant differences were found among the mean values of *τ*_1_ within this velocity range (Figure 12).

**Figure 13:**
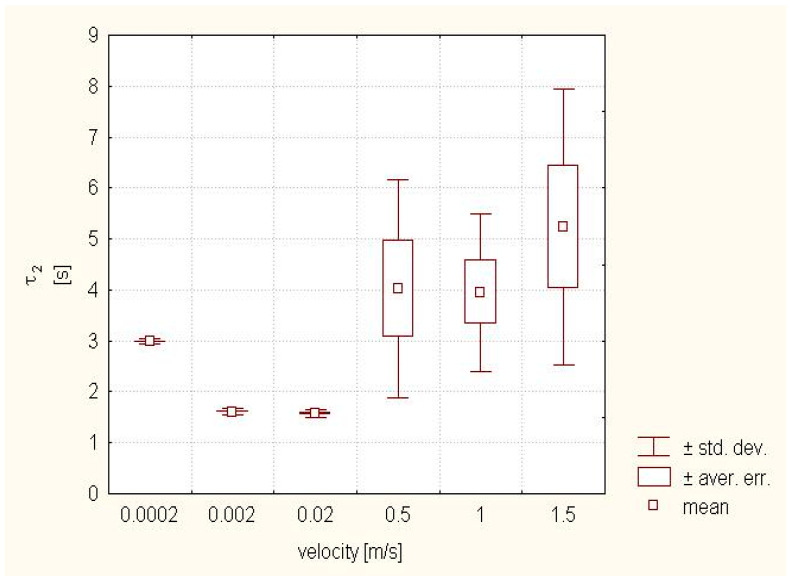
The relationship between relaxation time *τ*_2_ and deformation velocity.

**Figure 14:**
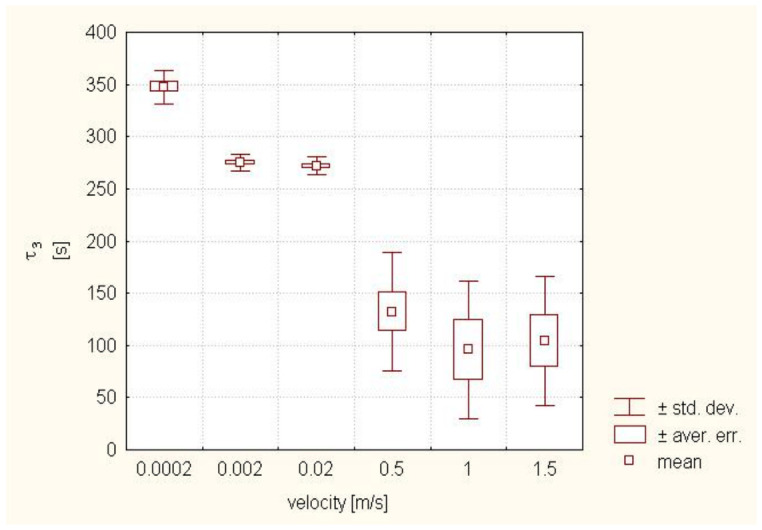
The relationship between relaxation time *τ*_3_ and deformation velocity.

The relaxation time *τ*_2_ was higher at deformation velocities of 0.5–1.5 m/s compared with quasi-static loading conditions (Figure 13). The relaxation time *τ*_3_ decreased with increasing deformation velocity, showing a trend similar to that observed for *τ*_1_. The *τ*_3_ values obtained under quasi-static loading conditions were substantially higher than those recorded under impact loading conditions (Figure 14).

## 4. Discussion

The tissues of fruit and vegetables exhibit complex mechanical properties resulting from their heterogeneous and anisotropic structure [19]. This structure consists of plant cells surrounded by cell walls and plasma membranes, containing vacuoles filled with cell sap, and interconnected through networks of intercellular connections within the middle lamella. As a result, plant tissues exhibit viscoelastic behavior, which can be described using rheological models, including the Maxwell model. The mechanical response of a material subjected to stress can be mathematically characterized by the elastic modulus (*E*), dynamic viscosity (*η*), and relaxation time (*τ*), defined as the ratio of viscosity to elastic modulus (*τ* = *η*/*E*). These parameters can be interpreted in relation to structure changes occurring in the cellular tissues during mechanical loading.

### 4.1. Elastic Modulus

The elastic modulus *E* represents the ability of tissue to respond immediately and elastically to an applied load. In plant tissues, this parameter is strongly related to the mechanical properties of cell walls and the level of cellular turgor pressure [20]. The cell wall, composed mainly of cellulose, hemicellulose and pectic substances, is the primary component responsible for tissue stiffness. A high content of ordered cellulose microfibrils and a high degree of pectin cross-linking within the middle lamella contribute to increased resistance to deformation.

Mechanical loads such as compression, impact, and vibration occurring during harvesting, transportation, and handling can cause cell deformation and microdamage within cell walls [21]. These processes reduce the effective stiffness of the cellular structure, which is reflected in rheological measurements as a decrease in the elastic modulus. Another factor contributing to the reduction in *E* is the loss of turgor pressure caused by plasma membrane damage, which results in cell sap leakage and a decrease in intracellular hydrostatic pressure.

### 4.2. Dynamic Viscosity

In the Maxwell model, dynamic viscosity *η* describes the resistance of a material to time-dependent deformation. In plant tissues, the viscous component is mainly associated with water movement within cells and intercellular spaces [22]. During mechanical deformation, various microstructural processes occur, including the sliding of cellulose microfibrils relative to each other [23], rearrangement of the pectin matrix, and movement of cell sap [24]. Damage to cell membranes affects their permeability and may promote the migration of cellular fluids into intercellular spaces. This process reduces the internal resistance of the tissue to deformation and may result in a decrease in the viscosity coefficient. Changes in viscosity can therefore reflect the degree of structural degradation of the tissue. As cell walls weaken and intercellular connections are disrupted, the ability of the tissue to dissipate mechanical energy decreases.

### 4.3. Relaxation Time

The relaxation time *τ* determines the rate of stress reduction in deformed material. In plant tissues, this parameter can be considered an indicator of structural stability and the ability of the tissue to redistribute mechanical stresses.

High relaxation time values indicate a well-preserved cellular structure in which cell walls and intercellular connections effectively transfer and dissipate stresses [25]. In contrast, a decrease in relaxation time observed after mechanical damage indicates tissue structure degradation [26]. This reduction may be associated with degradation of pectic substances in the middle lamella, weakening of intercellular adhesion, and enlargement of intercellular spaces [27,28].

These changes lead to reduced tissue cohesion, increased susceptibility to permanent deformation [29], and a shorter time required for stress relaxation.

Chen [30] applied a generalized Maxwell model consisting of elements characterized by different relaxation times. The element with a short relaxation time represents intercellular gases, which are capable of sustaining loads only for a very short period. The element with an intermediate relaxation time represented intercellular fluids, which flow more slowly than gases. The cellular structure itself was represented by an element with a very long relaxation time because the movement of intracellular fluids between cells occurs at a relatively slow rate. The water content of a potato tissue is approximately 80% [31], while air spaces account for only 3% of tissue volume [32]. Therefore, the contribution of gas flow within potato intercellular spaces can be considered negligible.

### 4.4. The Response of Plant Material to Different Loading Conditions

Plant cells respond differently to static and dynamic loading conditions. Under static loading, cells have sufficient time to redistribute water and partially compensate for applied stresses. In contrast, dynamic loading occurs over a much shorter time period, limiting the ability of cells to adapt and increasing the likelihood of structural damage [3].

Fruits and vegetables are highly hydrated materials, typically containing 80% to 95% water. In plant tissues, water can be divided into free and bound fractions, with free water occurring mainly in the cytoplasm and vacuoles. During impact loading, rapid cell deformation may result in cell rupture and the release of intracellular water, increasing moisture accumulation at the damaged surface. During low-velocity compression, gradual structural changes occur, including cell deformation and migration of free water from vacuoles [22].

In the 1980s, three major types of tissue damage caused by compression were identified: cell wall rupture, intercellular debonding, and cell relaxation associated with fluid migration from cells [33,34,35]. The dominant damage mechanism depends on the relative strength of cell walls and intercellular bonds, loading rate, product type, and tissue maturity.

Lin and Pitt [36] investigated the mechanical properties of potato tissue and showed that, under uniaxial compression at a constant strain rate, tissue failure could occur through either cell rupture or cell debonding, depending on the loading rate. When cell rupture dominated, higher strain rates reduced tissue strength because insufficient time was available for fluid migration from cells. When cell debonding was the dominant mechanism, strength reduction resulted from the extrusion of free cellular fluid, which became the primary resistance mechanism during loading.

Harker et al. [37] identified three mechanisms of damage in fruit tissues: cell rupture, cell fracture, and cell debonding. Fracture is characterized by the separation of cells along specific planes, while rupture involves cell bursting and collapse. Cell debonding involves separation between adjacent cells without severe damage or with only minor reductions in cell volume and deformation, while neighboring cells remain intact.

Different failure mechanisms of apple tissue have been reported depending on loading conditions. Under quasi-static loading, apple samples failed at a similar strain level, whereas under impact loading conditions, failure occurred at a relatively constant stress level [38].

Analysis of Maxwell model parameters provides valuable information about the structural characteristics of plant tissues and can be used to assess the severity of mechanical damage in fruit and vegetables. Changes in elastic modulus, dynamic viscosity, and relaxation time allow macroscopic mechanical properties to be related to processes occurring at the cellular level [39].

This approach enables the detection of early stages of tissue degradation that may not be apparent during visual evaluation of product quality [40]. Therefore, rheological analysis based on the Maxwell model represents a valuable tool for studying the effects of transportation, storage, and mechanical processing on the quality and shelf life of plant products.

## 5. Conclusions

The elastic modulus *E*_3_ was significantly higher under quasi-static than impact loading conditions. The relationship between *E*_3_ and deformation velocity closely corresponded to the changes observed in the minimum force, indicating that both parameters can effectively describe the state of potato tissue after deformation and provide information about the degree of cellular structure damage at the end of the stress relaxation test.

Relaxation times *τ*_1_ and *τ*_3_ decreased with increasing deformation velocity. Considering the relaxation time values in relation to the type of loading, it can be concluded that the greatest extent of tissue damage during impact occurred within the first 0.1 s and at the end of the test. Under quasi-static loading conditions, the highest rate of force decay was observed during the first 5 s of the relaxation process.

The maximum force response increased with increasing deformation velocity, confirming the viscoelastic nature of potato tissue. The difference between the initial maximum force and the minimum force recorded at the end of the test increased with increasing deformation velocity, indicating an intensification of destructive processes within the cellular structure at higher deformation velocity.

The parameters of the Maxwell model provide a useful tool for characterizing changes occurring in plant tissue. A decrease in elastic modulus, dynamic viscosity, and relaxation time may be associated with cell wall degradation, loss of turgor pressure, and disruption of cellular integrity, which are among the main mechanisms responsible for mechanical damage in fruit and vegetables.

Viscoelastic parameters can be applied to optimize the conditions for transportation, storage, and sorting of plant products. The results may support the selection of operating parameters for harvesting and processing equipment, such as working velocity and applied pressure. Furthermore, these parameters can serve as input data for numerical models, including finite element method (FEM) simulations used to predict the response of biological materials under different loading conditions.

## Data Availability

The original contributions presented in this study are included in the article. Further inquiries can be directed to the corresponding author.
